# Dynamic pressurization induces transition of notochordal cells to a mature phenotype while retaining production of important patterning ligands from development

**DOI:** 10.1186/ar4302

**Published:** 2013-09-17

**Authors:** Devina Purmessur, Clare C Guterl, Samuel K Cho, Marisa C Cornejo, Ying W Lam, Bryan A Ballif, Damien M Laudier, James C Iatridis

**Affiliations:** 1 Leni and Peter W. May Department of Orthopedics, Icahn School of Medicine at Mount Sinai, New York, NY 10029, USA; 2 Department of Biology, University of Vermont, Burlington, VT 05405, USA

## Abstract

**Introduction:**

Notochordal cells (NCs) pattern aneural and avascular intervertebral discs (IVDs), and their disappearance, is associated with onset of IVD degeneration. This study induced and characterized the maturation of nucleus pulposus (NP) tissue from a gelatinous NC-rich structure to a matrix-rich structure populated by small NP cells using dynamic pressurization in an *ex vivo *culture model, and also identified soluble factors from NCs with therapeutic potential.

**Methods:**

Porcine NC-rich NP tissue was cultured and loaded with hydrostatic pressure (0.5 to 2 MPa at 0.1 Hz for 2 hours) either Daily, for 1 Dose, or Control (no pressurization) groups for up to eight days. Cell phenotype and tissue maturation was characterized with measurements of cell viability, cytomorphology, nitric oxide, metabolic activity, matrix composition, gene expression, and proteomics.

**Results:**

Daily pressurization induced transition of NCs to small NP cells with 73.8%, 44%, and 28% NCs for Control, 1 Dose and Daily groups, respectively (*P *< 0.0002) and no relevant cell death. Dynamic loading matured NP tissue by significantly increasing metabolic activity and accumulating Safranin-O-stained matrix. Load-induced maturation was also apparent from the significantly decreased glycolytic, cytoskeletal (Vimentin) and stress-inducible (HSP70) proteins assessed with proteomics. Loading increased the production of bioactive proteins Sonic Hedgehog (SHH) and Noggin, and maintained Semaphorin3A (Sema3A).

**Discussion:**

NP tissue maturation was induced from dynamic hydrostatic pressurization in a controlled *ex vivo *environment without influence from systemic effects or surrounding structures. NCs transitioned into small nonvacuolated NP cells probably via differentiation as evidenced by high cell viability, lack of nitric oxide and downregulation of stress-inducible and cytoskeletal proteins. SHH, Sema3A, and Noggin, which have patterning and neurovascular-inhibiting properties, were produced in both notochordal and matured porcine NP. Results therefore provide an important piece of evidence suggesting the transition of NCs to small NP cells is a natural part of aging and not the initiation of degeneration. Bioactive candidates identified from young porcine IVDs may be isolated and harnessed for therapies to target discogenic back pain.

## Introduction

Low back pain is often associated with degeneration of the intervertebral disc (IVD). The condition is among the most common requiring physician visits, with enormous annual direct medical costs ($193 billion and rising) and also substantial lost productivity [1]. There is a need to develop novel biological treatments for IVD degeneration with the capacity to repair the IVD and to arrest the causes of discogenic pain. We believe that biological therapies for symptomatic disc degeneration will be more successful if they can recapitulate or otherwise utilize the important factors that exist during development, when the IVD is in homeostasis and anabolism outweighs catabolism.

The healthy and immature IVD is largely avascular and aneural with a highly gelatinous nucleus pulposus (NP) that is rich in notochordal cells (NCs) [2]. During growth and maturation, the IVD expands and the NP becomes more fibrous as the cellular niche is altered to include a greater percentage of matrix, increased hypoxia and reduced nutrient transport [3]. As the IVD degenerates it undergoes more significant alterations in structure, composition and cellular phenotype, with increased catabolism, inflammation and neurovascularization [4-8]. The nerves found in the degenerated IVD of back pain patients are considered one of the causes of discogenic pain [9].

NCs are of mesodermal origin and play an essential role in the formation and patterning of the spine and vertebrae during development. NCs contribute to the gelatinous nature of the healthy IVD via their high biosynthesis rates, and also because their complex cytoplasmic and vacuolated structure is believed to have osmoregulatory functions [10,11]. The function and disappearance of NCs during growth and aging are unclear. Studies have suggested that NCs die via apoptosis and are replaced by small nucleus pulposus cells (SNPCs) that migrate from the vertebral bodies through the endplate [12]. The ratio of large vacuolated NCs to small nonvacuolated NP cells in the NP region has long been known to decline with maturity of the human IVD [2]. Species that retain high proportions of large vacuolated NCs into adulthood (for example, rat, mouse, pig and rabbit) do not experience age-related disc degeneration as found in humans [8]. Consequently, the retention of NCs has long been postulated as a key factor in prolonging the longevity of a healthy spinal structure [9]. Recent lineage tracing studies using Sonic Hedgehog (SHH) and Noto have demonstrated that NCs and SNPCs are both derived from the embryonic notochord [13,14]. Risbud and Shapiro suggest that the reduction in the number of large vacuolated NCs in adult IVDs is associated with a shift in roles of the NCs as they become organizer cells or otherwise differentiate into smaller nonvacuolated NP cells required to maintain the NP matrix [15]. Recent studies by Sakai and colleagues have isolated progenitor cells (Tie2-positive and disialoganglioside-2-positive) from the NP of mouse and human IVDs, with multipotency and ability to differentiate into the mesenchymal lineages as well as reorganize the NP when implanted into immune-deficient mice. However, the relationship of these cells to NCs, their origin, or whether these cells express NC-like markers remains to be determined [16].

The mechanisms associated with transition and/or differentiation from a predominantly NC-rich to a predominantly SNPC-rich NP are largely unexplored. Studies suggest that NCs are very sensitive to their microenvironment as NC metabolism and viability are significantly affected by three-dimensional *in vitro *culture conditions, osmolarity, hypoxia and glucose levels [10,17-19]. Both injury and mechanical load influence maturation and transition of the young IVD to a more mature phenotype *in vivo*. For instance, a needle puncture induced sequential transformation from a chondrocyte-like IVD to a fibrous degenerate IVD, and static compressive load, induced a decline in NCs together with sclerosis of the endplate [18,20]. However, the mechanism by which the cellular composition changes from NC-rich NP to SNPC-rich NP (that is, via NC differentiation or NC death) is unclear.

Changes to the loading environment of the IVD can induce alterations suggestive of maturation. Yet *in vitro *studies have also shown that mechanical loading can have anabolic effects in terms of cell metabolism and biosynthesis. Daily cyclic hydrostatic loading at 3 MPa and 0.5 Hz for 30 minutes on rabbit NP cells increased protein synthesis and decreased matrix degradation [21], highlighting the increase in metabolic rate expected with stimulation from cyclic loading [22]. Healthy mature human NP cells also shifted towards an anabolic phenotype under dynamic hydrostatic loading, with increased expression of matrix proteins (SOX9 and collagen II) and decreased matrix metalloproteinase-3 (MMP3) expression [9].

An important function of NCs is the synthesis of bioactive soluble factors with the potential to stimulate and differentiate cells as well as to pattern the matrix of the NP. Soluble factors derived from NCs can stimulate the biosynthesis rates of NP cells and differentiate bone marrow-derived mesenchymal stem cells toward a healthy NP phenotype with an increase in proteoglycan production and expression of phenotypic markers characteristic of the healthy NP [23-26]. NCs also have the ability to protect other disc cells from apoptosis, matrix degradation and inflammation through the suppression of activated caspase 3, caspase 7/8, MMP3 and interleukin-6 expression [27,28]. We demonstrated recently that NCs produce soluble factors including several matricellular proteins that differentiate MSCs to a healthy NP phenotype with substantial amounts of GAG produced per cell. However, results also clearly indicated that soluble factors secreted from NCs were also very dependent upon NC culture conditions with retained cell matrix interactions in porcine NC-rich NP tissue promoting maximum GAG production [29]. SHH plays an important role in patterning of the IVD during development, and studies by Dahia and colleagues have highlighted a role for SHH signaling pathways in postnatal growth, differentiation and aging of the IVD [27,30]. Loss of SHH signaling has been associated with a decrease in differentiation markers such as SOX9 and Brachyury in NP cells as well as a reduction in proteoglycans. In addition to anabolic factors, the notochord expresses anti-neurogenic and anti-angiogenic factors during development including Semaphorin3A (Sema3A) and Noggin, which help to pattern and maintain the young aneural and avascular IVD. Such factors may be targeted to inhibit neurovascular ingrowth often associated with the discogenic pain because studies have shown that notochord-derived Sema3A and Noggin can repel neural or vascular ingrowth *in vitro *respectively [31,32]. Sema3A is also expressed by the mature IVD, in particular in the outer annulus fibrosus, and this decreases with degeneration of the IVD [33]. Whether these factors are expressed in the mature NP of species that retain NCs, or whether they are influenced by pressurization, remains largely unknown.

The first objective of this study is to initiate and characterize the cell and matrix changes associated with transition of NP tissue from one rich in NCs to one populated largely by SNPCs using dynamic hydrostatic pressurization in a controlled *ex vivo *organ culture model. Such a system allowed us to characterize and explore the mechanism of cellular transition in NP tissue without the influence of surrounding spinal structures or other systemic metabolic changes *ex vivo*. Culture models with *ex vivo *explants are useful tools to study the effects of micro-environmental conditions on native NP tissue [34]. The second objective was to measure the expression of the novel structure and symptom-modifying factors SHH, Sema3A and Noggin in response to daily dynamic loading during the maturation process. NCs were cultured in their native porcine NP tissue to retain cell structure and cell-matrix interactions, while *ex vivo *conditions provided a level of control in which the isolated effects of hydrostatic pressure could be examined. Hydrostatic pressure was applied to the gelatinous NP from young porcine IVDs since the IVD is subject to high amounts of cyclic pressurization *in vivo *and because cyclic loading is known to influence cell metabolism. Eight days was chosen based on previous studies and to observe changes at both the cell and molecular levels [21,35-37]. We hypothesized that daily dynamic load will induce maturation through differentiation of NCs to SNPCs while at the same time maintaining or enhancing the expression of bioactive factors in the NP.

## Materials and methods

### Dissection and isolation

Porcine spines (6 to 8 weeks of age) were obtained within 24 hours of death (Animal Facility Research 87 Inc., Boylston, MA, USA) and NC-rich NP tissue was dissected aseptically from three spines in total (*n *= 3) and divided into three groups with four discs from each spine per group: Control group (no pressurization), 1 Dose pressurization group, and Daily pressurization group. Lumbar and thoracic discs were treated equally irrespective of level to maximize the amount of tissue obtained per spine. Porcine NP tissue was cultured in 50 ml falcon tubes with 30 ml basal media consisting of high-glucose Dulbecco's modified Eagle's media, 1× insulin transferrin and selenium, salt solution (5 M NaCl/0.4 M KCl) [26,38,39] and 50 μg/ml ascorbic acid and incubated (1% oxygen, 5% carbon dioxide and 37°C) for 4 days prior to loading and during the 8-day loading period with the exception of when in the pressure vessel or in the water bath. Media were replaced every 4 days and media changes were performed in normoxic conditions.

### Hydrostatic loading protocol

**Figure 1:**
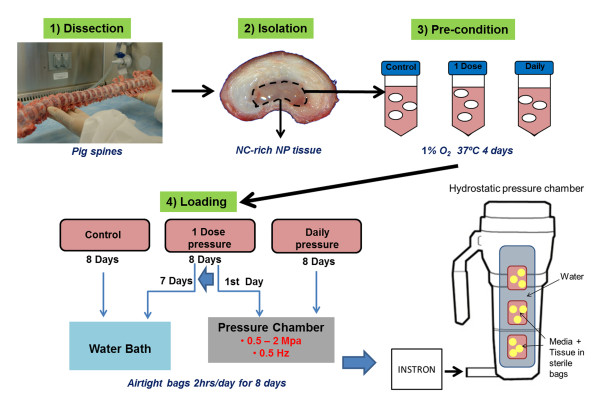
**Schematic of the tissue isolation and loading protocol**. Pig spines were dissected aseptically and the notochordal cell (NC)-rich nucleus pulposus (NP) tissue was isolated and preconditioned for 4 days in 1% oxygen, 5% carbon dioxide at 37°C in Dulbecco's modified Eagle's medium with 1× insulin transferrin and selenium. NP tissue was divided into three groups (Control group, 1 Dose pressurization group, Daily pressurization group) and placed in airtight Whirl-Pak bags (Nasco, Thermo Fisher Scientific, Waltham, MA, USA) for loading. The Control group was placed in a 37°C water bath for 2 hours every day for 8 days. The 1 Dose group was placed in a custom hydrostatic pressure chamber attached to an Instron 8511 servo hydraulic tension/compression system (Instron, Norwood, MA, USA) for 2 hours on 1 day with 0.5 to 2 MPa load at 0.1 Hz and then in the water bath for 2 hours each day thereafter [38]. The Daily pressurization group was placed in the chamber for 2 hours each day for 8 days with same loading regime as the 1 Dose group.

During the loading regime, NP tissue in 30 ml basal media from each group was transferred to individual sterile Whirl-Pak bags (Nasco, Thermo Fisher Scientific, Waltham, MA, USA) and sealed airtight. For the loaded groups, loading occurred in a custom-made hydrostatic pressure chamber attached to an Instron 8511 servo hydraulic tension/compression system (Instron, Norwood, MA, USA) (Figure 1) [40]. Control specimens were unloaded and placed in a 37°C water bath for 2 hours. The 1 Dose group was loaded (0.5 to 2 MPa at 0.1 Hz for 2 hours) on day 1 and on successive days placed in the water bath for 2 hours along with the Control group; the Daily loading group was loaded daily (0.5 to 2 MPa at 0.1 Hz for 2 hours) for eight days.

### Dependent variables

All groups were assessed for cell viability, metabolic activity, nitric oxide, real time quantitative reverse transcription-polymerase chain reaction (qRT-PCR) for NC phenotypic markers, histology for matrix accumulation, NC/NPC ratio analysis, immunohistochemistry for SHH and Sema3A, and proteomic analysis of soluble factors secreted into media, on day 9 (24 hours are completing the 8-day loading regime).

#### Cell viability

Cell viability in NP tissue was assessed using the Live/Dead assay (L3224; Invitrogen, Carlsbad, CA, USA). Briefly, approximately 2 mm×2 mm pieces of NP tissue (~0.5 mm thick) were incubated for 15 minutes in 10 ml high-glucose Dulbecco's modified Eagle's media containing 10 μl of 2 mM ethidium homodimer-1 stock and 20 μl of 4 mM calcein AM stock at 37°C protected from light. Following incubation, cell viability was assessed and images captured at 10× magnification using a fluorescent inverted Zeiss microscope (emission/excitation; calcein = 494/517 nm and ethidium homodimer-1 = 528/617 nm).

#### Metabolic activity

For metabolic activity - the (4,5-dimethylthiazol-2-yl)-2,5-diphenyltetrazolium bromide (MTT) assay was used and the wet weight of NP tissue was first measured and tissue digested as described previously [19,29]. Briefly, NP tissue was first digested with 0.2% protease (*Streptomyces griseous*, P5147, Sigma-Aldrich, St Louis, MO, USA) for 1 hour followed by 0.025% collagenase (*Clostridium histolyticum *type 1A, C2674; Sigma-Aldrich) for 18 hours at room temperature. This was followed by an additional digestion with a non-enzymatic cell dissociation solution (C1419; Sigma-Aldrich) for 2 hours. Cells were then incubated in a solution of 2 mg/ml MTT in high-glucose Dulbecco's modified Eagle's medium for 2 hours at 37°C, protected from light. After incubation, cells were pelleted, lysed in 200 μl dimethyl sulfoxide and absorbance was read on a plate reader at 570 nm [29]. Absorbance values for MTT were consistently normalized to wet weight of the tissue from which cells were digested because the DNA content (using the Picogreen assay) could not be assessed on the same tissue sample and cell numbers were too low for accurate counts on the hemocytometer.

#### Nitric oxide

To assess the amount of nitric oxide released into the media, the Griess reaction assay (G2930; Promega, Fitchburg, WI, USA) was performed as per the manufacturer's instructions. Briefly, the 0.1 M nitrite standard was serially diluted to give a reference curve of 100, 50, 25, 12.5, 6.25, 3.13 and 1.56 μM and both standards and media samples were pipetted into a 96-well plate. Then 50 μl sulfanilamide was added to all samples and incubated for 10 minutes followed by an additional 10-minute incubation period after adding *N*-1-napthylethylenediamine dihydrochloride solution. Absorbance was read between 520 and 550 nM.

#### Gene expression

Gene expression for NC phenotypic markers was assessed by first extracting RNA from NP tissue using the NucleoSpin^® ^RNA XS kit (740902.50; Macherey-Nagel, Düren, Germany) and transcribed to cDNA using the SuperScript^® ^VILO cDNA synthesis kit (11754-50; Invitrogen) as per the manufacturer's instructions. Real time qRT-PCR was run on an ABI 7500 (Applied Biosystems, Grand Island, NY, USA) using porcine-specific Taqman gene expression assays for the genes 18s (Hs03928985_g1), Aggrecan (Ss03374825_m1), Col1a1 and Col1a2 (Ss03373341_g1 and Ss03373345_g1), K18 (Ss03377383_u1), Brachyury (Ss03374655_m1) and Noggin (Ss03373959_s1). Cycle threshold (CT) values were normalized to both 18s and unloaded controls (Group 1) and 2^ΔΔCT ^was calculated as described previously [41].

#### Histology and immunohistochemistry

For histology and immunohistochemistry, NP tissue was encapsulated in Histogel, a tissue-processing medium to maintain tissue integrity, and then fixed in 10% zinc formalin for 48 hours. After fixation, samples were rinsed, dehydrated, and cleared using standard methods. After clearing, samples were infiltrated and polymerized with a hydrophobic acrylic resin. Then 4 μm thick sections were cut and mounted on silane-coated slides, and stained with either Safranin O/Fast green or prepared for immunohistochemistry. For Safranin O/Fast green staining, samples were first stained with hematoxylin for 7 minutes, washed and then stained with Fast green for 3 minutes. This step was followed by washing, agitation in 1% acetic acid for 15 seconds and staining in 0.1% Safranin O for 5 minutes. Samples were dehydrated through to xylene and mounted. For immunohistochemistry, sections were deplasticized and placed in a mild, non-heating Decal antigen-retrieval solution (HK089-5K; DAKO, Carpinteria, CA, USA) for 30 minutes. Sections were then rinsed with methanol followed by dH_2_O and treated with a protein block (X0909; DAKO) for 10 minutes. Rabbit monoclonal primary antibodies for SHH (ab53281; Abcam, Cambridge, MA, USA) and Sema3A (ab45376; Abcam) were diluted at 1:100 and 1:500, respectively, and were applied to sections overnight at 4°C. The antigens to which these antibodies bind demonstrate high sequence homology and cross-reactivity to porcine SHH and Sema3a respectively. A universal negative control rabbit IgG antibody was used for the negative control (N1699; DAKO). The next day, slides were washed with dH_2_O and then incubated in secondary antibody (rabbit-specific horseradish peroxidase conjugate, ab94710; Abcam) for 30 minutes at room temperature. DAB chromogen substrate was used to detect binding and sections were counterstained with methylene blue, dehydrated in xylene and mounted.

**Figure 2:**
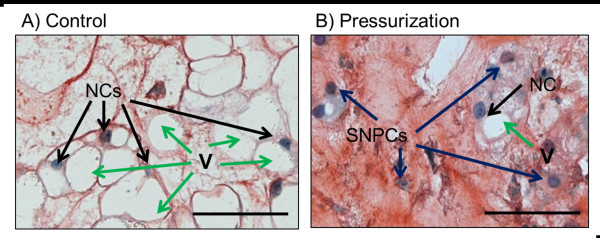
**Quantification of large notochordal cells versus small nucleus pulposus cells**. The number of notochordal cells (NCs) and small nucleus pulposus cells (SNPCs) in a total of nine images (3 images×3 spines used = 9) from each group (Control, 1 Dose and Daily) were counted and the percentage of NCs and percentage of SNPCs were calculated from the total number of cells in each image. **(A) **Control group image. **(B) **Pressurization group image. Black arrows, NCs; blue arrows, SNPCs. A large NC was defined as nuclei in direct contact and surrounded by large vacuoles, and an SNPC was defined as nuclei surrounded by matrix with no contact with vacuoles. V, vacuoles (green arrows). Scale bar = 50 μm.

Safranin O/Fast green-stained sections were also used to calculate the % of large NC versus small NPCs in each of the three groups. Brightfield images were captured at 10× magnification and nine images per group were assessed independently by two blinded reviewers. Single nuclei were counted as either large NC cells, defined as cells with nuclei surrounded by large cytoplasmic vacuoles, or small NPCs, defined as cells with nuclei surrounded by dense matrix without large cytoplasmic vacuoles (Figure 2).

#### Proteomics

Proteomic assessment, as described previously [29] (with methods modified to include serum-free basal media), was carried out on media samples from the Control (*n *= 3) and Daily pressurization (*n *= 3) groups. Briefly, media samples were run on SDS-PAGE gels and entire lanes were cut into five identical regions and subjected to in-gel reduction, alkylation and tryptic digestion [42]. Extracted peptides were subjected to liquid chromatography tandem mass spectrometry in a linear ion trap (LTQ)-Orbitrap hybrid mass spectrometer (Thermo Fisher Scientific, Waltham, MA, USA). Data were searched against the National Cancer Institute's nonredundant protein database using SEQUEST (NCI, Bethesda, MD, USA). Only proteins identified in conditioned media samples and not in basal media (that is, in the baseline medium prior to culture) and exclusively identified by peptides consistent with porcine origin were kept. Furthermore, only proteins where at least two peptides were identified from all six analyses were kept. The mean, standard deviation, mean peptide difference and statistical differences between proteins present in the unloaded and daily pressurization groups are shown in Additional files 1 and 2.

### Statistical analysis

One-way analysis of variance with a Newman-Keuls *post hoc *test was carried out on metabolic activity, gene expression using ΔΔCT values and assessment of cell differentiation (large versus small) using GraphPad Prism 3 (La Jolla, CA, USA) with *P *< 0.05 considered significant. A two-tailed Student's *t *test comparing the means of the unloaded and daily pressurization groups was conducted on the proteomic data assuming equal variance.

## Results

### Cell viability, metabolic activity and nitric oxide

**Figure 3:**
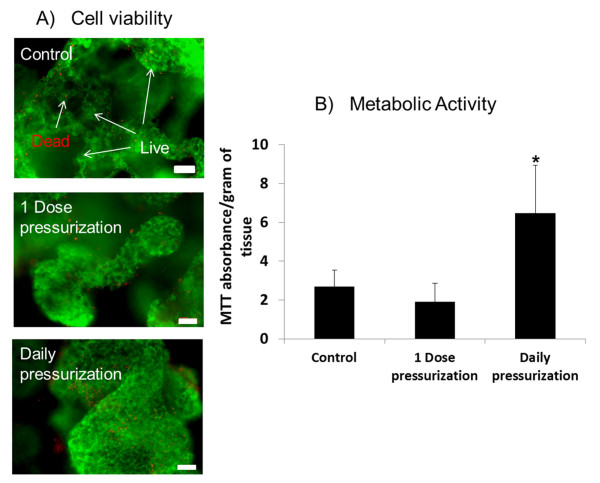
**Cell viability and metabolic activity in nucleus pulposus tissue with and without pressurization**. **(A) **Viability was assessed using calcein (green = live) and ethidium (red = dead) staining and showed no differences between groups, with the majority of the cells stained green indicating high viability (images captured on a fluorescent inverted microscope, bar =10 μm). **(B) **Metabolic activity was significantly increased for the Daily pressurization group (*P *< 0.05) as assessed by the (4,5-dimethylthiazol-2-yl)-2,5-diphenyltetrazolium bromide (MTT) assay compared with unloaded control and 1 Dose load groups.

Cell viability was assessed and no differences were observed between the unloaded Control, 1 Dose and Daily pressurization groups. Viability was high for all groups; calcein stained the majority of live cells green with few ethidium homodimer-1-stained red cells (Figure 3A). Assessment using the MTT assay demonstrated a significant increase in metabolic activity for the Daily pressurization group compared with the unloaded Control and 1 Dose of pressurization groups (Figure 3B) (*P *= 0.0275). Absorbance per gram of tissue was (mean ± standard deviation) 6.5 ± 2.5 for the Daily group compared with 1.9 ± 1.0 for the 1 Dose group and 2.7 ± 0.9 for the for unloaded Control group. Minimal nitric oxide was detected in the media with no differences between any of the groups; levels ranged within the noise at the bottom of the reference curve between 0.00 and 1.56 μM.

### Gene expression of matrix, notochordal cell phenotypic markers and bioactive ligands

**Figure 4:**
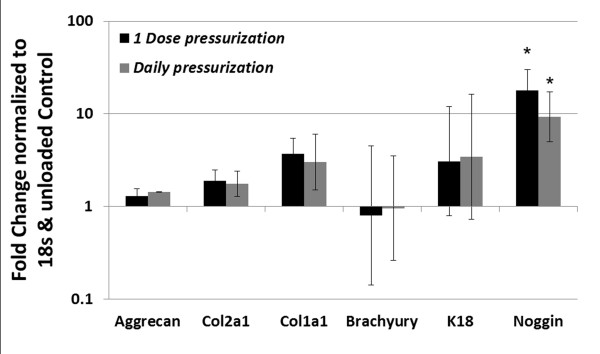
**Gene expression for notochordal cell phenotypic markers**. Quantitative reverse transcription real-time polymerase chain reaction was performed to assess fold-changes in gene expression in the1 Dose and Daily pressurization groups normalized to unloaded controls and housekeeping gene 18s. No significant changes were observed in Brachyury (T), K18, or matrix proteins (Aggrecan and Col1a1 and Col2a1), but there was a significant increase in Noggin expression with the Daily and 1 Dose load groups compared with the unloaded Control group (*P < 0.05).

When examining changes in gene expression, no significant changes were observed for matrix (Aggrecan, Col1a1 and Col1a2) and phenotypic (Brachyury and K18) genes for both the 1 Dose and Daily pressurization groups relative to the unloaded Control group (Figure 4). However, the bone morphogenetic protein antagonist and anti-angiogenic protein Noggin was upregulated with loading relative to the Control group; 18.0-fold and 9.2-fold for the 1 Dose and Daily groups, respectively (*P *= 0.0167).

### Histological staining and cell differentiation

**Figure 5:**
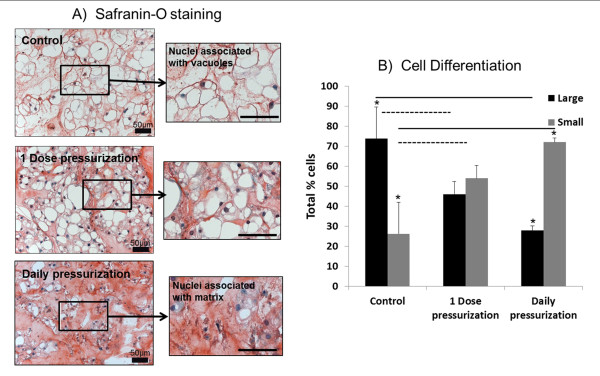
**Safranin O/Fast green staining of Control, 1 Dose and Daily pressurized nucleus pulposus tissue**. **(A) **Nucleus pulposus (NP) tissue demonstrated increased accumulation of proteoglycan (red staining) with pressurization and this was greatest with the Daily pressurization group. **(B) **A notable change in cell morphology was also observed with loading, with fewer large notochordal cells (NCs) and increased numbers of small nucleus pulposus cell (SNPCs) with load compared with the Control group (black bars). The 1 Dose pressurization group also demonstrated significant differences from the Control group (grey bars) and there were significant differences between NCs and SNPCs in the Control and Daily pressurization groups (*). To quantify this, the number of large NCs versus SNPCs was calculated based on the criteria shown in Figure 2 (*P *< 0.0002).

Safranin O/Fast green staining of NP tissue demonstrated increased intensity of proteoglycan staining and matrix accumulation in both pressurization groups and was most prominent in the Daily pressurization group (Figure 5A). These same histological samples were used to quantify the number of large NC versus SNPC as a percentage of the total number of cells for each group (Figure 5B). The number of NCs and SNPCs in unloaded control tissue was 73.8% and 26.2%, respectively. The 1 Dose group shifted to 44% NCs and 56% SNPCs. The Daily pressurization group had a significant decrease in NCs to 28% with 72% SNPCs compared with the unloaded Control group (*P *= 0.0002).

### Immunohistochemistry for Sonic Hedgehog and Semaphorin 3A

**Figure 6:**
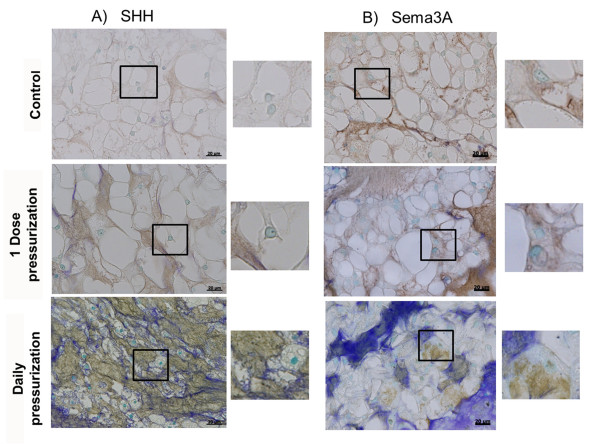
**Immunohistochemistry for Sonic Hedgehog and Semaphorin 3A expression**. Sonic Hedgehog (SHH) positive staining in nucleus pulposus (NP) tissue and a qualitative increase was observed with pressurization associated both with the cell and matrix **(A)**. Semaphorin 3A (Sema3A) expression was also detected in NP tissue in the cells and matrix, but no differences in expression were observed with load **(B)**.

Immunostaining for the developmental ligand SHH was observed in all groups with the most abundant staining in the pressurization groups compared with control (Figure 6A). SHH stained the matrix and cell nuclei. Staining for the neuronal inhibitor Sema3A was also observed in all groups with no difference from pressurization (Figure 6B). Sema3A immunostaining was observed mostly in the matrix with some staining in cell nuclei from all groups. Negative control samples for Rabbit IgG isotype staining were negative.

### Proteomic analysis

Proteomic analysis of media samples from unloaded Control and Daily pressurization groups identified a number of proteins of interest, with the majority of proteins identified being extracellular (see Additional files 1 and 2). Given the semi-quantitative nature of the proteomic analysis, relatively few proteins were expected to show statistical differences; yet the proteins that were significantly (*P *< 0.05) different between the Control and Daily pressurization groups, with peptide differences >2, are illustrated in Table 1. These included Vimentin with a peptide difference of 4 between the Daily load and Control groups, Heat shock protein 70 kDa with a difference of 2.7, phosphoglycerate mutase-1 with a difference of 2.3, α_1_-antitrypsin with difference 2.7, and glucose-6-phosphate isomerase with a difference of 9.3 (a trend with *P *= 0.07). Fibronectin demonstrated the greatest peptide difference of 33.3 between the Control and Daily pressurization groups although this was not significant (see Additional files 1 and 2).

**Table 1:** Proteomic analysis of conditioned media samples from Daily dynamic and unloaded Control groups demonstrating significant peptide differences between groups >2

Protein	Control		Daily load		Peptide difference	*P *value	Function
				
	Mean number of peptides	SD	Mean number of peptides	SD			
Vimentin	4	1.73	0	0	-4.00	0.02	Cytoskeletal protein: responsive to mechanical load [23,40,55]
Heat shock protein 70 kDa	3.7	0.6	1	1.6	-2.7	0.02	Notochordal cells have lower levels than small nucleus pulposus cells][47]
Phosphoglycerate mutase-1	2.3	1.2	0.0	0	-2.3	0.02	Glycolytic pathway [56]
α_1_-Antitrypsin	3.3	1.2	0.7	0.6	-2.7	0.05	Inhibits serine proteases and can bind aggrecanases [52]
Glucose-6-phosphate isomerase	15.66	6.50	6.33	1.53	-9.33	0.07	Glycolytic pathway [57]

Proteomics identified five proteins that were differentially expressed between loaded and daily loaded groups. SD, standard deviation.

## Discussion

This study demonstrated that exposure of NC-rich NP tissue to daily dynamic hydrostatic pressurization in an *ex vivo *culture model induced changes representative of maturation of the IVD with a decreased percentage of NCs and increased GAG-rich matrix accumulation. The expression of bioactive proteins with the capacity to maintain an avascular and aneural IVD was maintained or increased during this maturation process, providing evidence to suggest that loss of NCs is probably not an initiator of IVD degeneration but may be a normal part of aging without implications for development of painful conditions. We propose that the mechanism by which the notochordal NP transitions to an IVD populated largely by SNPCs is differentiation of NCs to SNPCs, rather than NC loss by cell death, because we observed no differences in cell viability or nitric oxide levels between groups, and also noted decreased cell stress proteins (that is, Heat shock protein 70 kDa) with loading.

**Figure 7:**
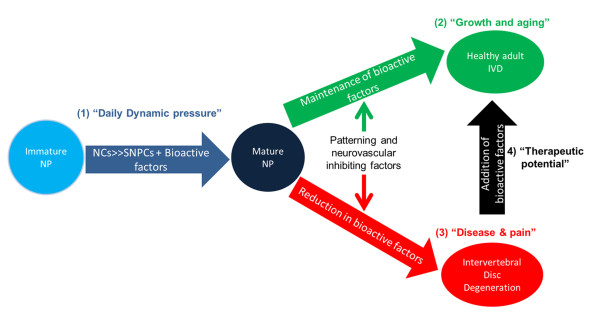
**Conceptual model of maturation and painful intervertebral disc degeneration**. Results suggest that loss of notochordal cells (NCs) and maintenance of bioactive ligands is associated with growth and aging of the intervertebral disc (IVD) and not related to disease and pain. **(1) **Daily dynamic pressure induces differentiation of NCs into small nucleus pulposus cells (SNPCs) and increased matrix accumulation. The maintenance or increase in the production of soluble bioactive factors associated with patterning and neurovascular inhibition suggest such maturation of the nucleus pulposus (NP) can be considered a natural part of growth and aging **(2)**, and results in the formation of the healthy adult IVD. However, a decrease in bioactive factors may result in neurovascular ingrowth and predispose the IVD to disease and pain **(3)**. Such reductions may occur due to injury or microenvironmental changes. Furthermore, we hypothesize that that these bioactive factors have therapeutic potential and can be isolated and used to treat painful IVD degeneration **(4)**.

We introduce a new conceptual model (Figure 7) of maturation and painful IVD degeneration in which dynamic pressurization induces maturation of NP tissue, differentiation of NCs to SNPCs, and maintenance of developmental patterning and neurovascular inhibiting factors (SHH, Sema3A and Noggin). However, we consider this to be a natural part of growth and aging, resulting in formation of the healthy mature adult IVD, and suggest that a reduction in NCs alone may not necessarily lead to IVD degeneration. Yet to validate such a claim, comparisons are required in more mature animals that lose NCs with age, such as human and bovine species [43,44]. The increases in matrix accumulation and fibronectin and the changes in cell population and metabolism do coincide with some changes that have been observed in growth, differentiation, and aging models in the mouse [20,45]. We propose that a reduction or loss of these bioactive factors from injury, changes in IVD microenvironment or genetic predisposition leads to the onset of painful disc disease. Finally, we speculate that that addition of these patterning and neurovascular inhibiting factors may have therapeutic potential to treat painful IVD degeneration, and further studies are warranted to validate this concept.

### Maturation and differentiation

Results suggest that NCs differentiate into SNPCs under dynamic pressurization conditions and differentiation is greater when the cells are subjected to daily load. We observed greater proteoglycan accumulation together with increased numbers of SNPCs in the Daily pressurization group. Previous studies by Kim and colleagues suggested that NCs die via apoptosis and chondrocyte-like cells from the endplate migrate and replenish the cellular niche of the NP [12,46]. Our *ex vivo *culture system demonstrated that the NC loss and increase in SNPCs is probably not due to cell death and that maturation in response to dynamic pressurization may occur *in vitro *without the presence of the annulus fibrosus or endplate; however, further studies *in vivo *are necessary to validate the role of surrounding spinal structures. The high cell viability between our groups together with the minimal nitric oxide in the culture media and decreases in heat shock proteins support the concept that NCs do not disappear as a result of cell death but likely differentiate into SNPCs. We suggest that examining NC-rich NP tissue prior to culture would also have been a useful comparison, in particular with respect to validating our culture system and the parameters assessed. In our unloaded controls we observed cell ratios of NCs to SNPCs similar to previous studies including limited cell matrix in the NC-rich NP [2,29,47,48].

Lineage studies using SHH and Noto as developmental markers have demonstrated recently that NCs and SNPCs are both derived from the embryonic notochord, which provides credence to the idea that cells from surrounding non-notochordal structures do not populate the NP as it matures and SNPCs are probably derived from NCs [13,14]. This is supported by the expression of similar phenotypic markers Brachyury (T) and K18 in both NCs and SNPCs although these markers are reportedly reduced in the aging and degenerate IVDs [45,49]. We observed no significant changes in these markers in our groups. *In vitro *two-dimensional culture of NCs isolated from the rabbit NP have shown that NCs can undergo changes in cell morphology and structure [50], providing evidence from the literature that NCs have the ability to differentiate into multiple cell types. The results from our work together with the literature strongly support the hypothesis that NC differentiation to SNPCs is the mechanism of cellular transition during maturation. Such transition could be further characterized by labeling or transfecting NCs and markers assessed post maturation in SNPCs to track individual cells during such differentiation, although this was beyond the scope of our study.

Our proteomics data of conditioned media samples from the Daily load and unloaded Control groups further elucidates the process of NC differentiation to SNPCs. Daily pressurization significantly decreased the abundance of a number of intracellular proteins in conditioned media such as Vimentin, a mechanically responsive cytoskeletal intermediate filament protein, and glycolytic proteins such as phosphoglycerate mutase 1 and glucose-6-phosphate isomerase including heat shock proteins that was unexpected. We speculate that the presence of increased numbers of intracellular proteins in unloaded Control media samples may be associated with small membrane vesicles called exosomes, which can release intracellular contents of the cell into the extracellular space and are involved in a number of biological processes; however, this is only a theory and requires further testing [51]. We propose that a decrease in these proteins in conditioned media from explants treated with Daily loading may be associated with a transition in function of NCs to a newly differentiated state. Decreases in α_1_-antitrypsin, a protease inhibitor that interacts with aggrecanase-1, may be associated with the increased proteoglycan turnover observed at the matrix level as the NP tissue matures [52]. Proteomic analysis is by nature semi-quantitative, so these data are largely used to support and interpret the changes we identified at both the cell and matrix levels.

### Biosynthesis and expression of structure and symptom-modifying factors

Dynamic pressurization has long been known to increase cell biosynthesis including increasing IVD cell metabolism [21,37]. Daily dynamic pressurization significantly increased metabolic activity in NP tissue compared with the 1 Dose and Control groups in this study. It is possible that the 1 Dose group exhibited an increase in metabolic activity following the day 1 pressurization event, which returned to control levels by the end of the experiment. Increased matrix and proteoglycan accumulation in NP of the Daily pressurization group was strongly demonstrated by Safranin-O/Fast green staining. However, no significant changes for matrix proteins were observed at the gene level although all proteins demonstrated a trend of upregulation. The differences between gene and protein level changes are probably a consequence of either temporal or kinetic differences between the two molecular profiles. It would therefore have been of interest to also assess gene expression earlier in the loading protocol (24 or 48 hours post stimulation or after the 1 Dose of load) where we would expect to observe more pronounced changes at the gene level, and this is a limitation of the current study.

During growth and development, the notochord expresses a number of ligands involved in patterning of the spine and vertebrae. Important in this study is the concept that such ligands involved in patterning and formation of the IVD may be useful for therapeutics. SHH is such a ligand, essential for the formation of the NP [53], and more recent studies by Dahia and colleagues have shown an important role for SHH in postnatal growth of the IVD including regulation of the transforming growth factor beta pathway. SHH maintains expression of phenotypic makers such as Brachyury as well as expression of matrix proteins [27]. In our culture system, SHH was expressed in notochordal and matured NP, and was associated with large NCs, SNPCs and matrix. Expression was increased with pressurization, supporting a role for SHH in increasing matrix accumulation and transition to the mature IVD. However, further clarification is necessary to identify downstream signaling targets such as PTCH1 and GLI1 as well as to assess mRNA rather than proteins to distinguish the cells that are synthesizing SHH and those that are responding to it. We hypothesize that SHH may also be a suitable structure-modifying factor that could be utilized for restoring NC function and/or promoting an NP phenotype over hyaline cartilage phenotypes.

The notochord also synthesizes factors that contribute to the avascular and aneural nature of the immature IVD, including the neuronal inhibitor Sema3A and the bone morphogenetic protein antagonist and anti-angiogenic factor Noggin. Both Sema3A and Noggin have been shown to inhibit neurite outgrowth and vascularization during spinal development respectively, and Sema3A has also been shown to reduce pain behavior when injected into an *in vivo *model of chronic injury to the sciatic nerve [54]. We observed expression of Sema3A in our NP tissue and expression was maintained between groups. However, an increase in expression of Noggin at the mRNA level with cyclic pressurization suggests that these bioactive molecules produced by NCs could also be functional as symptom-modifying therapeutics for treatment of painful IVDs. For these studies, we assessed the immunohistochemical staining qualitatively since the SHH and Sema3A expression was detected in both the cells and matrix of NP tissue, so that counting the percentage of positive cells did not accurately represent the extent of positive staining. Worth noting is that similar patterns of positive staining were noted in NP tissue across all samples within the same treatment groups. Proteomic analysis identified matricellular proteins Tenascin and Clusterin as demonstrated previously by our group but was unable to detect bioactive factors such as SHH, Sema3A or Noggin [29]. This may be a consequence of matricellular proteins being of higher molecular weight and can therefore be more easily detected by proteomic analysis, while detection of smaller ligands such as SHH and Sema3A were more amenable to antibody techniques such as immunostaining.

The concept that such soluble factors are not cell-type specific was suggested previously in porcine studies showing that conditioned media from NP and annulus fibrosus cells had similar effects on annulus fibrosus cells [23]. While static loading *in vivo *has been shown to induce changes associated with NC and maturation in the rabbit IVD, we suggest that dynamic hydrostatic pressurization is a more physiological load to study such cellular transitions since it can enhance biosynthesis, metabolism and also increase the expression of bioactive factors. We have now documented a tissue culture model system in which controlled NC differentiation to SNPCs can be induced. Further characterization of how the specific matrix components of this tissue change during this transition is warranted. The identification of the mechanism of differentiation using specific markers is also important, but the fact that NCs and SNPCs share a common origin as well as many of same markers makes this a difficult challenging task that requires future work.

## Conclusions

This is the first study to show that daily dynamic pressurization of porcine NP tissue in an *ex vivo *culture model induces NP tissue maturation with transition from a NC-rich to SNPC-rich tissue and increased proteoglycan accumulation. Our data suggest that the loss of NCs with maturation and aging is association with differentiation of NCs to SNPCs. However, dynamic loading can maintain or increase the expression of important structure- (that is, SHH) and symptom-modifying factors (for example, Sema3A and Noggin). This knowledge suggests that the transition and differentiation of NCs to SNPCs may not be an initiator of IVD degeneration but is a natural process of growth and maturation in the IVD of species that lose their NCs. We hypothesize that the causes of initiation and progression of disease and pain involve reductions in bioactive patterning and neurovascular inhibiting factors. Painful IVD degeneration is therefore complex and multifactorial, likely involving larger changes to the IVD than the loss of NCs such as injurious loading and altered microenvironment conditions occurring in the context of genetic susceptibility.

## Abbreviations

IVD: intervertebral disc; MTT: (4,5-dimethylthiazol-2-yl)-2,5-diphenyltetrazolium bromide; NC: notochordal cell; NP: nucleus pulposus; Sema3A: Semaphorin 3A; SHH: Sonic Hedgehog; SNPC: small nucleus pulposus cell.

## Competing interests

The authors declare that they have no competing interests.

## Authors' contributions

DP was involved in the study design, performed experimental work, data analysis and interpretation, and drafted the manuscript. CCG participated in the study design, experimental work, data analysis and helped to draft the manuscript. SKC participated in the study design, data analysis and interpretation. MCC contributed to the experimental work, data analysis and interpretation. BAB and YWL performed the SDS-PAGE and proteomic assessment of media groups, data analysis and write-up, and helped with data interpretation. DML participated in the experimental work and data analysis. JCI secured funding, contributed to the study design, organized and executed the study, and helped with data analysis and interpretation including drafting the manuscript. All authors read and approved the manuscript.

## Supplementary Material

Additional file 1 **Table S1 presenting the full table of proteomics data for intracellular protein**. All peptides identified related to intracellular proteins in NCCM from the Control and daily pressurization groups. Proteins identified by mass spectrometry from the Control and Daily load samples. These two samples were chosen as we anticipate that these two groups would yield the greatest magnitude difference in effects. Proteins were identified as described in Methods. Protein symbols including hyperlinks are provided. Proteins are categorized into either secreted/extracellular or primarily intracellular proteins. Three Control and Daily Load samples are shown with the total number of peptides identified from each indicated protein. Also provided are the average number of peptides identified from Control or Daily Load samples, the standard deviation (STD), the difference (DL-C) and results of a Student's *t *test comparing the two means. Click here for file

Additional file 2 **Table S2 presenting the full table of proteomics data for extracellular proteins**. All peptides identified related to extracellular proteins in NCCM from the Control and daily pressurization groups. Proteins were identified using the same method as for intracellular proteins. Click here for file
