# Supplementary material for: Dynamic pressurization induces transition of notochordal cells to a mature phenotype while retaining production of important patterning ligands from development
Source: Arthritis Res Ther. 2013 Sep 17;15(5):R122. doi: 10.1186/ar4302 (PMC3978427; doi:10.1186/ar4302)
Supplement: Additional file 1 — Table S1 presenting the full table of proteomics data for intracellular protein. All peptides identified related to intracellular proteins in NCCM from the Control and daily pressurization groups. Proteins identified by mass spectrometry from the Control and Daily load samples. These two samples were chosen as we anticipate that these two groups would yield the greatest magnitude difference in effects. Proteins were identified as described in Methods. Protein symbols including hyperlinks are provided. Proteins are categorized into either secreted/extracellular or primarily intracellular proteins. Three Control and Daily Load samples are shown with the total number of peptides identified from each indicated protein. Also provided are the average number of peptides identified from Control or Daily Load samples, the standard deviation (STD), the difference (DL-C) and results of a Student's t test comparing the two means. [file ar4302-S1.PDF]

| Protein Symbol | Protein Name Secreted/Extracellular | Control (C) | | | | | Daily Load (DL) | | | | | DL-C | |
|---|---|---|---|---|---|---|---|---|---|---|---|---|---|
| | | #1 | #2 | #3 | Average | STD | #1 | #2 | #3 | Average | STD | Diff | T-Test |
| A1AT | Alpha-1-anti-trypsin | 4 | 4 | 2 | 3.3 | 1.2 | 2 | 0 | 0 | 0.7 | 1.6 | -2.7 | 0.05 |
| ACAN | Aggrecan core protein | 0 | 2 | 5 | 2.3 | 2.5 | 1 | 2 | 5 | 2.7 | 1.8 | 0.3 | 0.87 |
| AMYC | pancreatic alpha-amylase-like | 0 | 6 | 0 | 2.0 | 3.5 | 0 | 1 | 0 | 0.3 | 2.2 | -1.7 | 0.46 |
| CAH3 | Carbonic anhydrase 3 | 3 | 4 | 5 | 4.0 | 1.0 | 5 | 3 | 6 | 4.7 | 1.6 | 0.7 | 0.56 |
| CHAD | Chondroadherin-like (Predicted) | 12 | 6 | 5 | 7.7 | 3.8 | 4 | 3 | 2 | 3.0 | 3.2 | -4.7 | 0.11 |
| CILP | Cartilage intermediate layer protein 1 | 0 | 4 | 0 | 1.3 | 2.3 | 0 | 0 | 0 | 0.0 | 1.5 | -1.3 | 0.37 |
| CLUS | Clusterin | 26 | 11 | 4 | 13.7 | 11.2 | 16 | 19 | 7 | 14.0 | 6.9 | 0.3 | 0.97 |
| CO1A2 | collagen, type I, alpha 2 | 1 | 0 | 0 | 0.3 | 0.6 | 2 | 7 | 2 | 3.7 | 2.3 | 3.3 | 0.12 |
| CO2A1 | Collagen, type I, alpha 1 (Predicted) | 11 | 13 | 6 | 10.0 | 3.6 | 9 | 7 | 11 | 9.0 | 3.1 | -1.0 | 0.70 |
| CO4 | Complement C4 | 0 | 3 | 1 | 1.3 | 1.5 | 0 | 2 | 2 | 1.3 | 1.0 | 0.0 | 1.00 |
| CO6A1 | Collagen type VI alpha-1 (Predicted) | 3 | 2 | 2 | 2.3 | 0.6 | 2 | 4 | 8 | 4.7 | 2.2 | 2.3 | 0.26 |
| CO6A2 | Collagen type VI alpha-2 (Predicted) | 1 | 2 | 5 | 2.7 | 2.1 | 3 | 2 | 4 | 3.0 | 1.3 | 0.3 | 0.81 |
| CO6A3 | Collagen type VI alpha-3 (Predicted) | 2 | 4 | 3 | 3.0 | 1.0 | 2 | 2 | 16 | 6.7 | 4.9 | 3.7 | 0.48 |
| COBA2 | Collagen, type XI alpha 2 | 2 | 4 | 0 | 2.0 | 2.0 | 2 | 3 | 5 | 3.3 | 1.5 | 1.3 | 0.41 |
| COBA2-like | Collagen, type XI alpha 2-like | 5 | 2 | 1 | 2.7 | 2.1 | 2 | 1 | 0 | 1.0 | 1.5 | -1.7 | 0.28 |
| CSPG2 | Versican | 2 | 0 | 1 | 1.0 | 1.0 | 0 | 0 | 0 | 0.0 | 0.7 | -1.0 | 0.16 |
| ECM1 | Extracellular matrix protein 1 | 1 | 4 | 2 | 2.3 | 1.5 | 1 | 2 | 0 | 1.0 | 1.2 | -1.3 | 0.27 |
| FINC | Fibronectin (Predicted) | 59 | 81 | 88 | 76.0 | 15.1 | 74 | 99 | 155 | 109.3 | 39.2 | 33.3 | 0.26 |
| FMOD | Fibromodulin | 25 | 14 | 7 | 15.3 | 9.1 | 11 | 14 | 15 | 13.3 | 5.4 | -2.0 | 0.73 |
| IBP5 | Insulin-like growth factor-binding protein 5-like (Predicted) | 0 | 0 | 0 | 0.0 | 0.0 | 1 | 2 | 1 | 1.3 | 0.8 | 1.3 | 0.02 |
| IBP7 | Insulin-like growth factor-binding protein 7 | 0 | 0 | 0 | 0.0 | 0.0 | 0 | 1 | 1 | 0.7 | 0.5 | 0.7 | 0.12 |
| ICA | serotransferrin isoform 1 | 2 | 0 | 0 | 0.7 | 1.2 | 0 | 0 | 1 | 0.3 | 0.7 | -0.3 | 0.68 |
| NID2 | nidogen-2 | 0 | 0 | 2 | 0.7 | 1.2 | 0 | 0 | 2 | 0.7 | 0.9 | 0.0 | 1.00 |
| O75339 | cartilage intermediate layer protein 1-like (predicted) | 0 | 0 | 0 | 0.0 | 0.0 | 0 | 2 | 0 | 0.7 | 0.7 | 0.7 | 0.37 |
| OPTC | Opticin | 6 | 6 | 1 | 4.3 | 2.9 | 3 | 2 | 5 | 3.3 | 1.8 | -1.0 | 0.62 |
| OSTP | Osteopontin | 11 | 11 | 7 | 9.7 | 2.3 | 7 | 8 | 8 | 7.7 | 2.8 | -2.0 | 0.22 |
| PGS2 | Decorin | 1 | 2 | 2 | 1.7 | 0.6 | 2 | 2 | 2 | 2.0 | 0.6 | 0.3 | 0.37 |
| PLOD1 | Procollagen-lysine,2-oxoglutarate 5-dioxygenase 1 | 0 | 1 | 4 | 1.7 | 2.1 | 0 | 0 | 0 | 0.0 | 1.4 | -1.7 | 0.24 |
| PRELP | Prolargin | 2 | 0 | 0 | 0.7 | 1.2 | 0 | 0 | 0 | 0.0 | 0.8 | -0.7 | 0.37 |
| Q5VU13 | V-set and immunoglobulin domain-containing protein | 0 | 0 | 0 | 0.0 | 0.0 | 2 | 0 | 0 | 0.7 | 0.7 | 0.7 | 0.37 |
| SMOC1 | SPARC-related modular calcium-binding protein 1 | 2 | 2 | 0 | 1.3 | 1.2 | 2 | 1 | 1 | 1.3 | 0.7 | 0.0 | 1.00 |
| SPRC | SPARC-like isoform 2 | 2 | 2 | 1 | 1.7 | 0.6 | 2 | 3 | 2 | 2.3 | 0.7 | 0.7 | 0.23 |
| TARSH | Target of Nesh-SH3 | 3 | 1 | 3 | 2.3 | 1.2 | 0 | 0 | 4 | 1.3 | 1.5 | -1.0 | 0.54 |
| TENA | Tenascin | 0 | 2 | 4 | 2.0 | 2.0 | 0 | 1 | 0 | 0.3 | 1.4 | -1.7 | 0.24 |
| TIMP2 | TIMP metallopeptidase inhibitor 2 | 2 | 2 | 1 | 1.7 | 0.6 | 1 | 2 | 2 | 1.7 | 0.6 | 0.0 | 1.00 |
