# Supplementary material for: Dynamic pressurization induces transition of notochordal cells to a mature phenotype while retaining production of important patterning ligands from development
Source: Arthritis Res Ther. 2013 Sep 17;15(5):R122. doi: 10.1186/ar4302 (PMC3978427; doi:10.1186/ar4302)
Supplement: Additional file 2 — Table S2 presenting the full table of proteomics data for extracellular proteins. All peptides identified related to extracellular proteins in NCCM from the Control and daily pressurization groups. Proteins were identified using the same method as for intracellular proteins. [file ar4302-S2.PDF]

Protein Symbol	Protein Name	Control (C)					Daily Load (DL)					DL-C	
	Intracellular	#1	#2	#3	Average	STD	#1	#2	#3	Average	STD	Difference	T-Test
ACTB	Actin, cytoplasmic 1	16	15	12	14.3	2.1	5	13	11	9.7	5.0	-4.7	0.16
ACTN4	Alpha-actinin-4	1	0	0	0.3	0.6	1	0	4	1.7	1.3	1.3	0.35
ANXA2	Annexin A2	33	27	15	25.0	9.2	19	25	26	23.3	7.6	-1.7	0.79
ANXA5	Annexin A5	10	11	12	11.0	1.0	9	13	11	11.0	3.7	0.0	1.00
ANXA8	Annexin A8	0	1	0	0.3	0.6	1	1	0	0.7	0.5	0.3	0.52
EF11	Eukaryotic translation elongation factor 1 alpha 2	0	1	0	0.3	0.6	0	0	1	0.3	0.4	0.0	1.00
EF1A0	Elongation factor 1-alpha 1	0	2	0	0.7	1.2	0	0	0	0.0	0.8	-0.7	0.37
ENO1	Enolase	8	5	6	6.3	1.5	3	1	6	3.3	2.5	-3.0	0.15
EZRI	Ezrin	2	3	0	1.7	1.5	0	0	0	0.0	1.2	-1.7	0.13
G6PI	Glucose-6-phosphate isomerase	22	16	9	15.7	6.5	6	5	8	6.3	6.1	-9.3	0.07
GAPDH	Glyceraldehyde-3-phosphate dehydrogenase	3	3	3	3.0	0.0	4	4	3	3.7	1.2	0.7	0.12
GDIR	Rho GDP-dissociation inhibitor 1	2	2	2	2.0	0.0	2	1	1	1.3	0.8	-0.7	0.12
GELS	Gelsolin	0	3	6	3.0	3.0	2	9	4	5.0	2.7	2.0	0.50
GNAI1	Guanine nucleotide-binding protein G(i) subunit alpha-1 (Predicted)	2	1	0	1.0	1.0	0	1	0	0.3	0.7	-0.7	0.37
GTR1	Glucose transport protein	1	0	0	0.3	0.6	1	0	0	0.3	0.4	0.0	1.00
H2A1	PREDICTED: histone H2A.x-like	1	1	0	0.7	0.6	0	0	0	0.0	0.5	-0.7	0.12
HS70A	Heat shock 70kDa protein 8	4	4	3	3.7	0.6	1	0	2	1.0	1.6	-2.7	0.02
KIF4A	Kinesin family member 4A	0	0	0	0.0	0.0	5	0	0	1.7	1.8	1.7	0.37
KPYK	Pyruvate kinase isozymes M1/M2 (Predicted)	4	2	3	3.0	1.0	2	2	9	4.3	2.5	1.3	0.61
LDHA	L-lactate dehydrogenase A chain	7	3	3	4.3	2.3	2	3	4	3.0	1.6	-1.3	0.41
PGAM1	Phosphoglycerate mutase 1	3	3	1	2.3	1.2	0	0	0	0.0	1.3	-2.3	0.02
PRDX1	Peroxiredoxin-1	2	1	1	1.3	0.6	0	0	1	0.3	0.7	-1.0	0.10
Q13025	Enhancer of mRNA-decapping protein 4	0	0	0	0.0	0.0	2	0	0	0.7	0.7	0.7	0.37
Q29568	Phosphopyruvate hydratase	1	1	0	0.7	0.6	0	0	2	0.7	0.7	0.0	1.00
RAB10	RAB10, member RAS oncogene family	2	2	0	1.3	1.2	2	0	0	0.7	0.9	-0.7	0.52
TBA1	Tubulin, alpha 1d	2	0	4	2.0	2.0	0	0	0	0.0	1.5	-2.0	0.16
VAT1	Synaptic vesicle membrane protein VAT-1 homolog	2	1	0	1.0	1.0	2	1	3	2.0	0.9	1.0	0.29
VIM1/VIME	Vimentin	5	2	5	4.0	1.7	0	0	0	0.0	2.2	-4.0	0.02
VINC	Vinculin	0	0	2	0.7	1.2	0	0	0	0.0	0.8	-0.7	0.37
